# Decreased Galectin-9 and Increased Tim-3 Expression Are Related to Poor Prognosis in Gastric Cancer

**DOI:** 10.1371/journal.pone.0081799

**Published:** 2013-12-10

**Authors:** Jing Jiang, Mei-Shan Jin, Fei Kong, Donghui Cao, Hong-Xi Ma, Zhifang Jia, Yin-Ping Wang, Jian Suo, Xueyuan Cao

**Affiliations:** 1 Division of Clinical Epidemiology, First Hospital of Jilin University, Changchun, China; 2 Division of Pathology, First Hospital of Jilin University, Changchun, China; 3 Department of Gastric and Colorectal Surgery, First Hospital of Jilin University, Changchun, China; Institute of Cancerology Gustave Roussy, France

## Abstract

**Introduction:**

Galectin-9 (Gal-9) induces adhesion and aggregation of certain cell types and inhibits the metastasis of tumor cells. T-cell immunoglobulin–and mucin domain-3–containing molecule 3 (TIM-3) plays a pivotal role in immune regulation. The aim of this study is to investigate Gal-9 and TIM-3 alterations in gastric cancer and their prognostic values.

**Methods:**

Gal-9 and Tim-3 expression was evaluated using a tissue microarray immunohistochemistry method in 305 gastric cancers, of which 84 had paired adjacent normal samples. Cell lines SGC-7901, BGC-823, MGC-803, MKN45 and GES-1 were also stained. Correlations were analyzed between expression levels of Gal-9 and Tim-3 protein and tumor parameters or clinical outcomes.

**Results:**

Gal-9 and Tim-3 stained positive on tumor cells in 86.2% (263/305), and 60.0% (183/305) patients with gastric cancer, respectively. Gal-9 expression was significantly higher in cancer than in normal mucosa (*P*<0.001). Reduced Gal-9 expression was associated with lymph-vascular invasion, lymph node metastasis, distant metastasis and worse TNM staging (*P* = 0.034, *P* = 0.009, *P* = 0.002 and *P* = 0.043, respectively). In contrast, Tim-3 expression was significantly lower in cancer than in control mucosa (*P*<0.001). Patients with lymph-vascular invasion had higher expression levels of Tim-3 (*P*<0.001). Moreover, multivariate analysis shows that both high Gal-9 expression and low Tim-3 expression were significantly associated with long overall survival (*P* = 0.002, *P* = 0.010, respectively); the combination of Gal-9 and Tim-3 expression was an independent prognostic predictor for patients with gastric cancer (RR: 0.43; 95%CI: 0.20–0.93). *H.pylori* infection status was not associated with Gal-9 and Tim-3 expression (*P* = 0.102, *P* = 0.565).

**Conclusion:**

The results suggest that expression of Gal-9 and Tim-3 in tumor cells may be a potential, independent prognostic factor for patients with gastric cancer. Gal-9 and TIM-3 may play an important part in the gastric carcinogenesis.

## Introduction

Gastric cancer remains the fourth most commonly diagnosed cancer and is the second leading cause of cancer related deaths worldwide [1]. Galectins are a group of proteins that bind β-galactosides through evolutionarily conserved sequence elements of the carbohydrate recognition domain (CRD)[2], [3]. The galectin family contributes to neoplastic transformation, tumor cell survival, tissue invasion and metastasis of several types of cancer, including gastric cancer [4]. Previous studies showed that the expression level of galectin-3 was significantly higher in the gastric carcinomas compared to adjacent normal tissue. Galectin-3 and galectin-4 may be useful tumor markers for gastric cancers with respect to tumor progression and potentiality of lymph node metastasis especially in certain histological types of gastric cancer [5], [6]. Galectin-9 (Gal-9) is a new member of the galectin protein family [7]. In recent years, an important role has emerged for the Gal-9 in health and disease [8]. In normal physiology, Gal-9 seems to be a pivotal modulator of T-cell immunity by inducing apoptosis in specific T-cell subpopulations. Because these T-cell populations are associated with autoimmunity, inflammatory disease, and graft rejection, it was postulated that application of exogenous Gal-9 may limit pathogenic T-cell activity [9], [10]. In addition, Gal-9 is localized in the cytoplasm and on the cell surface of cells and seems to be released from tumor cells [11]. In solid tumors, the available studies suggested that Gal-9 has a tumor-suppressor function, with loss of Gal-9 being closely associated with increased metastasis and high recurrence [12], [13], [14], [15]. Kadowaki et al also showed that Gal-9 signaling can prolong survival by inducing macrophages to differentiate into plasmacytoid dendritic cell-like macrophages in a mouse lung-cancer model [16]. These datas presented that Gal-9 may be a possible prognostic factor with anti-metastatic potential. However, expression of Gal-9 has not yet been investigated in gastric cancer.

T-cell immunoglobulin– and mucin domain-3–containing molecule 3(Tim-3) is a T helper type 1 (Th1)–specific cell surface molecule that seems to regulate Th1 responses and the induction of peripheral tolerance [17], [18]. Recently, aberrant Tim-3 expression was reported in melanoma cells, contributing to the low adhesion ability of tumor cells and promoting the survival of the tumor [19]. Zhuang et al also demonstrated the significant role of Tim-3 expression in tumor cells as an independent prognostic factor for patients with non–small cell lung cancers [20]. Zhu C et al showed that Gal-9 is the Tim-3 ligand, and suggested that the Tim-3–Gal-9 pathway may have evolved to ensure effective termination of effector Th1 cells [21]. To investigate the relationship between Tim-3–Gal-9 pathway and progress of gastric cancer, the Gal-9 and Tim-3 protein expression was investigated in 305 patients with gastric cancer, of which 84 cases had paired adjacent normal tissue samples. Associations of the protein expression with patient clinical characteristics and prognosis were also explored in this study.

## Methods

### Study Populations

Three hundred and five patients including 231 men and 74 women with gastric cancer who underwent surgery between August 2000 and December 2010 at First Hospital of Jilin University were enrolled in this study. The patients did not receive any treatment before the surgical operation. The diagnosis of gastric cancer was made on the basis of morphologic and immunohistochemical findings evaluated by two independent pathologists (MSJ and YPW). Gastric cancers were removed by surgical excision; adjacent normal gastric epithelial samples were also collected from 84 patients for comparison. Patient ages ranged from 32 to 87 years, with a median age of 64 years. Written informed consent was obtained from all of the patients and the study protocol was approved by the ethics committee of the First Hospital of Jilin University.

### Cell culture

Human gastric cancer cell lines SGC-7901, BGC823, MGC803, MKN45 were acquired from Department of Gastric and Colorectal Surgery, First Hospital of Jilin University. The immortalized human gastric mucosal cell line GES-1 provided by the Cancer Hospital of Beijing University. Cells were cultured in RPMI-1640 medium containing 10% heat-inactivated fetal bovine serum (FBS) and 100 ng/ml each of penicillin and streptomycin in an incubator (50 ml/l CO2) at 37°C. Medium on the cells was changed every 2-3 days. Cells in logarithmic growth phase were collected for further experiments.

### Immunohistochemistry

In brief, the sections (4 µm in width) from the tissue microarry (TMA) blocks were deparaffinized and dehydrated, heat-induced epitope retrieval was conducted by immersing slides in 10 mmol citrate buffer (pH 6.0) and boiling the buffer for 10 mi in a pressure cooker. After blocking with 3% H_2_O_2_, All sections were incubated overnight at 4°C with anti human galectin-9 polyclonal antibody (1∶250 diluted, ab69630, Abcam, UK) and anti human Tim-3 polyclonal antibody (8 µg/ml, AF2365, R&D Systems, Minneapolis, USA), 3,3-Diaminobenzidine (DAB) was employed as a chromogen, and the sections were counterstained with hematoxylin. For negative controls, tumor slides were treated with the IgG isotypes from rabbit (normal rabbit IgG, sc-2027, Santacruz, USA) and goat (normal goat IgG,sc-2342, Santacruz, USA) to replacement of primary antibody, all negative controls demonstrated negligible background stain. Cell lines (SGC-7901, BGC-823, MGC-803,MKN45 and GES-1) were routinely cultured, gastric cancer cell pellets were embedded in paraffin and processed as tissue slides. Gastric cancer cell lines were stained with the same method.

The stained slides were evaluated by two independent pathologists (MSJ and YPW), who were blinded to clinical data and outcome, The widely accepted semi-quantitative analysis (HSCORE system) was used to assess staining intensity and percentages of the cells stained with a specific magnitude of intensity. The HSCORE was calculated using the following equation: HSCORE = ∑Pi(i) (i =  0,1,2,3, Pi = 0∼100%). i means the intensity of staining, i.e. no staining  = 0, weak staining  = 1, moderate staining  = 2 and strong staining  = 3. Pi represents percentages of stained cells with intensities varying from 0 to 100. Therefore, the HSCORE ranges from 0 to 300, HSCORE>0 is considered as positive staining and HSCORE  = 0 is considered as a complete negative staining. The discrepant results of staining intensity from two pathologists were resolved by reviewing the cases together and agreeing on the scores, and the discrepant results of percentages of stained cells were resolved by calculating the means of the scores.

### Western blotting

To confirm the accuracy of semi-quantification by immune-histology, The Western blotting were administrated in gastric cancer cells lines. The cells were lysed with cold lysis buffer and centrifuged at 12000 g for 10 minutes, loading buffer were mixed with cell lysates and boiled for 5 minutes at 95°C. 10 µg total protein was loaded into 10% discontinuous SDS polyacrylamide gel for electrophoresis and then transferred onto polyvinylidene fluoride membranes. Membranes were blocked in 5% bovine serum albumin and incubated with primary antibodies (anti Gal-9 and anti Tim-3) overnight at 4°C in Tris buffered saline with 0.1% Tween20. After washing, membranes were incubated in horseradish peroxidase-conjugated antibodies for 1 hour at room temperature. Membranes were then incubated and western blotting images were taken and analyzed. β-actin was used as the internal control and all densitometric values were normalized against the relative β-actin value.

### Determination of *H.pylor*i infection

Among 305 gastric cancer cases, blood samples were collected from 104 patients for the examination of *H.pylor*i infection before the surgical operation. 4 ml of fasting blood samples were left at room temperature for 30 minutes and then centrifuged. The serum was drawn and stored at −80°C. Serum immunoglobulin (Ig) G antibodies to *H.pylori* were detected by *H.pylor*i-IgG enzyme-linked immunosorbent assay (ELISA) kit (Biohit, Helsink, Finland). The antibody titres were defined by optical density (OD) values according to the manufacturer's protocol and titres higher than the cut off value of 30EIU were considered as positive for *H.pylori* infection. The kit quality control samples showed coefficient of variation of 4.5% for *H*.*pylori* infection examination.

### Statistics analysis

The HSCORE of Gal-9 and Tim-3 expression are presented as median (interquartile). The Wilcoxon matched-pairs signed-rank test and Mann-Whitney U test or Kruskal-Wallis H test were used when comparing paired groups and two independent groups respectively. The Kaplan-Meier method was used to estimate the overall survival rate, and survival differences were analyzed using the log-rank test. The Cox proportional hazards model was used to calculate the relative risks (RRs) and corresponding 95% confidence intervals (95%CIs) after adjusting by age (scale variable), gender (nominal variable), lymph-vascular invasion (nominal variable) and TNM staging (scale variable). All statistical tests were two-tailed and *P*-values less than 0.05 were considered to be statistically significant. All analyses were performed using the SPSS software package 18.0 (SPSS Inc. USA).

## Results

### Expression of Gal-9 and Tim-3 in gastric cancer and gastric cancer cell lines

**Figure 1:**
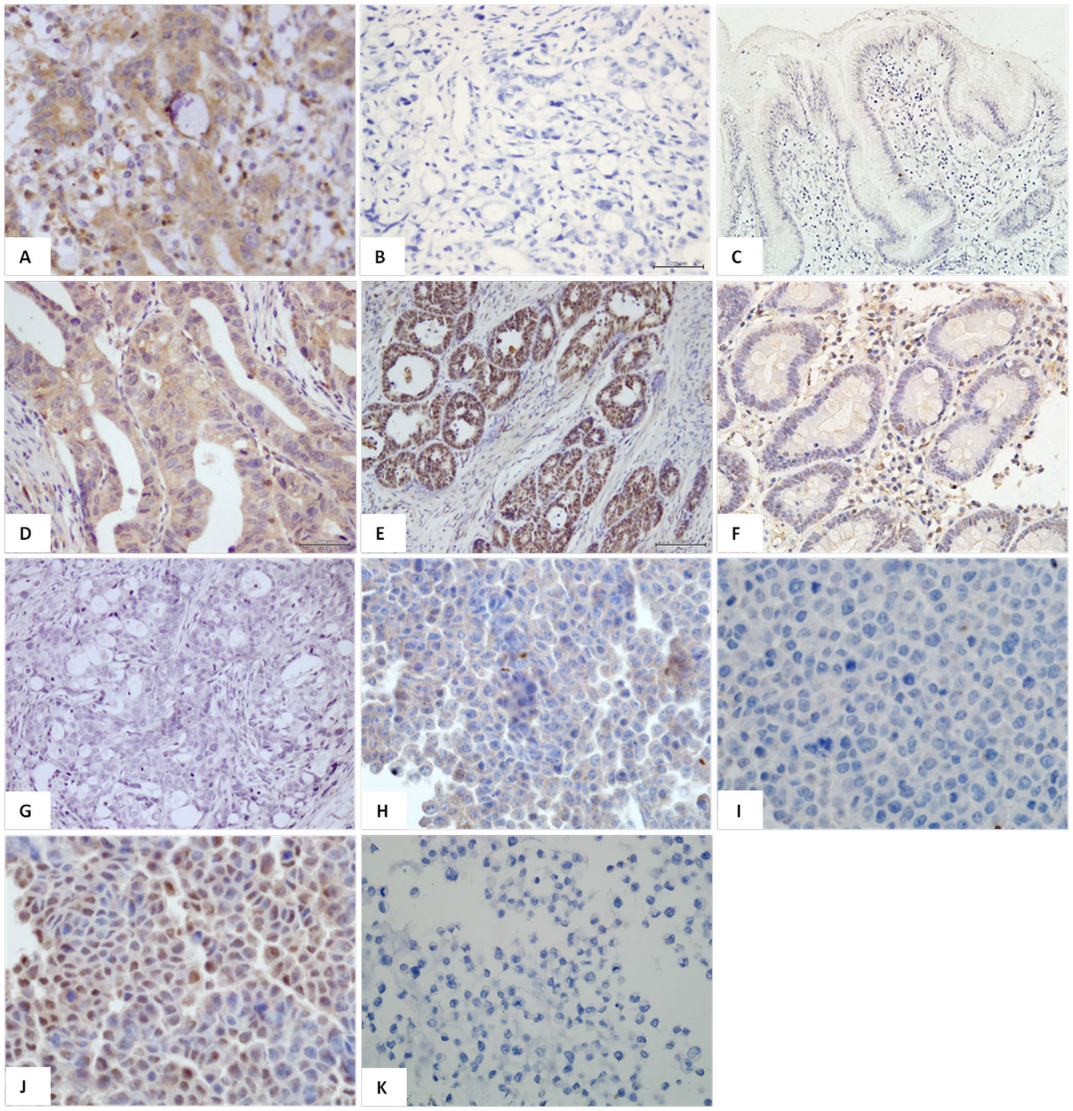
Immunohistochemical analysis indicated that most of the cancer cells showed cytoplasmic Gal-9 immunostaining(A), but a small number of gastric cancer cells(B) and gastric normal mucusa showed negative immunostaining for Gal-9 (C). Gastric tumor cells showed a clear cytoplasmic(D),nuclear positivity (E), or negative for Tim-3 immunostaining (G). (F) showed a weak Tim-3 positive staining in gastric normal mucusa. Cell lines SGC-7901, BGC-823 and MGC-803 and GES-1 showed weak cytoplasmic Gal-9 immunostaining (H, BCG-823), but MKN45 showed negative immunostaining for Gal-9(I). Four gastric cancer cell lines also showed strong cytoplasmic and nuclear positivity for Tim-3 staining (J, BCG-823 cell line). However, GES-1 cells showed negative staining for Tim-3(K). Original magnifications, x200 and x400.

Immunohistochemical analysis showed that Gal-9 and Tim-3 stained positive on tumor cells in 86.2% (263/305), 60.0% (183/305) patients with gastric cancer, respectively. Most of the cancer cells in gastric cancer tissue showed potent cytoplasmic Gal-9 immunostaining, but a small number of gastric cancer cells showed negative immunostaining for Gal-9 (Figure. 1A, 1B,1C). Gastric tumor cells showed a clear cytoplasmic and nuclear positivity for Tim-3 immunostaining (Figure. 1D, 1E,1F,1G).

Among 84 paired samples, Gal-9 positive staining was found in 68/84 (81.0%) gastric cancer and in basal cells of 51/84 (60.7%) normal mucosa, respectively (*P* = 0.004). Conversely, Tim-3 positive staining was found 55/84 (65.5%) in gastric cancer and 72/84 (82.7%) in normal mucosa, respectively (*P* = 0.002). In addition, cell lines SGC-7901, BGC-823and MGC-803 and GES-1 showed weak cytoplasmic Gal-9 immunostaining(Figure. 1H), but MKN45 showed negative immunostaining for Gal-9(Figure. 1I). Four gastric cancer cell lines also showed strong cytoplasmic and nuclear positivity for Tim-3 staining (Figure. 1J). However, GES-1 cells showed negative staining for Tim-3(Figure.1K).

**Figure 2:**
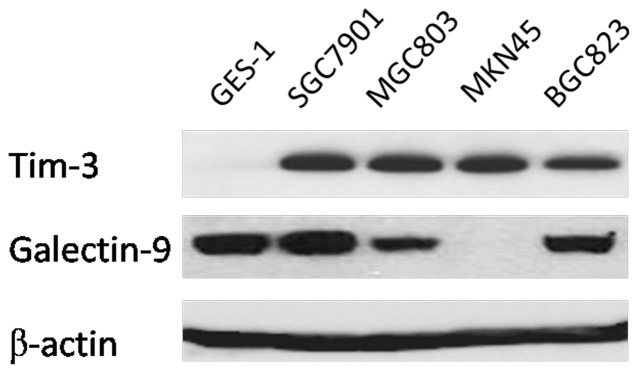
The Western blotting results of cell lines were consistent with the Immunohistochemical analysis of gastric cancer tissues. Note that both Tim-3 and Gal-9 were detected in gastric cancer cell lines MGC803,SGC7901, and BGC823, absence of Gal-9 activation in MKN45 cells, and negative of Tim-3 immunostaining in GES-1 were also confirmed.

The detection of Gal-9 and Tim-3 activation was validated from the same cells by Western blotting, which is shown in Figure 2.

**Figure 3:**
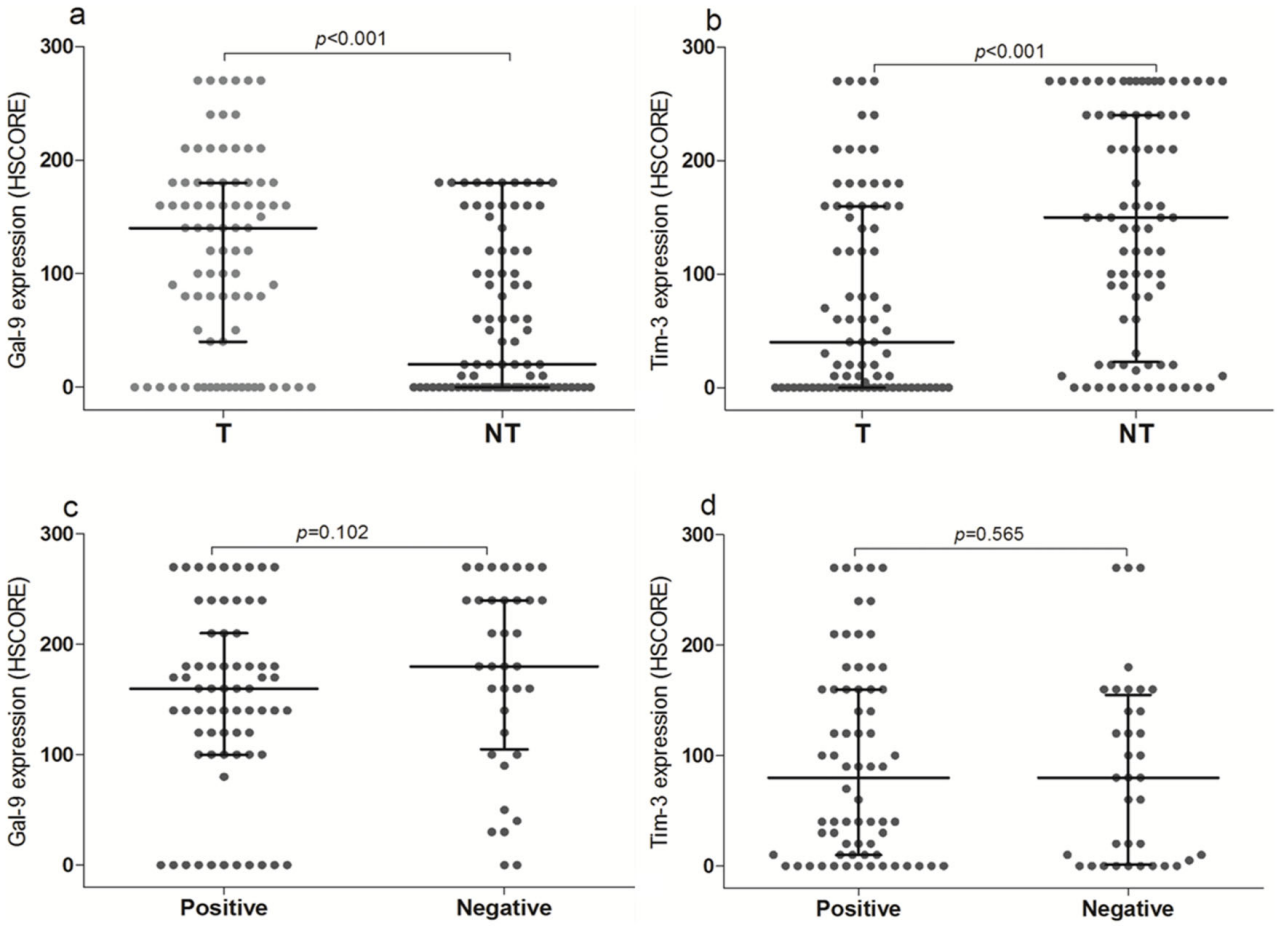
Gal-9 expression was significantly higher in gastric tumor tissues (T) compare to those in paired adjacent non-tumor tissues(3a), and Tim-3 HSCORE in cancer lesion was significantly lower than those in normal mucosa(3b). However, no differences of Gal-9(3c) or Tim-3 expressions (3d) were found between patients with *H*.*pylori* infection (Positive) and without *H.pylori* infection (Negative).

Gal-9 expression in cancer lesions was significantly higher than those in normal mucosa (Median HSCORE: 140 vs 20; *P*<0.001. Figure 3A). Tim-3 HSCORE in cancer lesions was significantly lower than those in normal mucosa (Median HSCORE: 40 vs 170; *P* < 0.001. Figure 3B).

### Gal-9 and Tim-3 expression levels and *H.pylor*i infection

From 104 gastric cancer patients tested for *H.pylor*i antibodies, the total positive rate of *H.pylor*i infection was 65.4%. In the age subgroup analysis, the *H.pylor*i infection rate was higher in the 45–65 years group, compared to the younger than 45 years old groups, or older than 65 years old groups. The results showed no correlation between *H.pylor*i infection and the Gal-9 and Tim-3 expression (*P* = 0.102, *P* = 0.565). (Figure 3C, 3D).

### Correlation of Gal-9 and Tim-3 expression with clinicopathologic parameters

As shown in table 1, HSCORE of Tim-3 was significantly higher in male patients than female patients (*P* = 0.010). Tim-3 expression levels were positively correlated with age (*P* = 0.004), however Gal-9 expression levels were negatively correlated with age (*P* = 0.049). There were significant differences in Gal-9 and Tim-3 expression levels among subgroups of venous infiltration, in patients with venous infiltration, the median HSCORE of Gal-9 was significant lower (*P* = 0.034) and Tim-3 was significant higher (*P*<0.001). According to the TNM stages, higher HSCORE was observed in patients of stage I tumors compare to other stages (*P* = 0.043). The significantly higher HSCORE of Gal-9 was found in patients without lymph node metastasis (*P* = 0.009), and in patients without distant metastasis (*P* = 0.022). We also analyzed the Gal-9 and Tim-3 expression according to tumor differentiation (High, Middle and Low) and depth of invasion (T1-T4), no significant differences were found. The clinical characteristics of subjects are summarized in table 1 in detail.

**Table 1:** Galectin-9 and Tim-3 expressions in gastric cancer according to clinicopathologic parameters.

	Gal-9 HSCORE	*P* value	Tim-3 HSCORE	*P* value
	Median(quantile range)		Median(quantile range)	
Gender				
Male (n = 231)	160(100–240)	0.362	40(0–150)	0.010
Female(n = 74)	160(115–240)		0(0–80)	
Age(years)				
≤60(n = 130)	180(120–240)	0.049	5(0–90)	0.004
>60(n = 175)	160(80–240)		60(0–160)	
TNM stage				
I (n = 25)	240(120–255)	0.043	20(0–120)	0.406
II(n = 51)	170(120–240)		20(0–120)	
III(n = 195)	160(100–210)		20(0–120)	
IV(n = 34)	140(100–172)		50(0–160)	
Differentiation				
Well (n = 6)	125(65–218)	0.795	10(0–82.5)	0.108
Middle(n = 114)	160(100–240)		60(0–160)	
poor (n = 185)	160(100–240)		20(0–120)	
Lymph-vascular invasion				
Absent(n = 142)	170(120–240)	0.034	0(0–90)	<0.001
Present(n = 163)	160(100–210)		60(0–160)	
Depth of invasion				
T1(n = 9)	240(90–270)	0.236	70(0–90)	0.407
T2(n = 38)	180(120–240)		0(0–125)	
T3(n = 225)	160(100–240)		20(0–140)	
T4(n = 33)	140(62–210)		60(0–160)	
Lymph metastasis				
N0(n = 67)	210(140–270)	0.009	20(0–120)	0.872
N1(n = 93)	160(120–180)		40(0–140)	
N2(n = 78)	160(78–240)		30(0–145)	
N3(n = 67)	140(100–180)		20(0–140)	
Distant metastasis				
Negative(n = 268)	160(100–240)	0.022	20(0–120)	0.058
Positive(n = 37)	140(90–170)		70(0–160)	
Survival				
Survival(n = 183)	180(120–240)	0.017	20(0–120)	0.067
Death(n = 122)	160(100–180)		60(0–140)	

### High Gal-9 expression and negative Tim-3 expression were significantly associated with long overall survival

**Figure 4:**
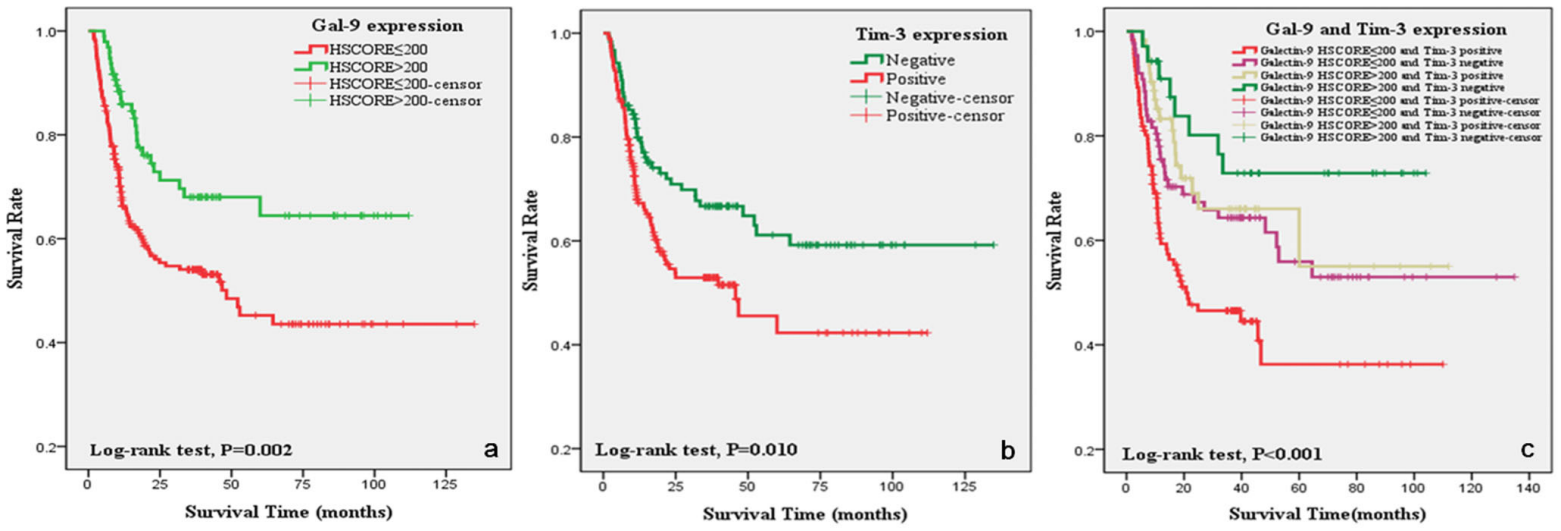
Multivariate analysis shows that high Gal-9 expression (4a), low Tim-3 expression(4b), and both Gal-9 HSCORE>200 and Tim-3 negative expression (4c) were significantly associated with long overall survival in gastric cancer patients.

Follow-up information was available for all 305 patients, covering periods ranging from 3 to 135 months (median 40 months). No patient died of postoperative complications within 30 days of the beginning of the study period, and 120(39.3%) patients died during the follow up. Kaplan-Meier survival curves were plotted according to the HSCORE (Negative, HSCORE≤100, HSCORE≤200 and HSCORE>200). The overall survival rate was significantly higher in Gal-9 HSCORE>200 group than other groups (Log-rank test, *P* = 0.011). As survival curves of negative group, HSCORE≤100 group and HSCORE≤200 group were similar, we combined them for further analysis, and Kaplan-Meier survival curve were shown in Figure.4A(*P* = 0.002). Patients with Tim-3-negative tumors had longer overall survival. The same results were obtained when all positive expression patients were combined (Figure.4B, Log-rank test, *P* = 0.01). Kaplan-Meier survival curves were also plotted according to the Gal-9 and Tim-3 co-expression, the overall survival rate of patients who were Gal-9 HSCORE>200 and Tim-3 negative was significantly higher, and patients who were Gal-9 HSCORE≤200 and Tim-3 positive was significantly lower (Figure.4C, Log-rank test, *P*<0.001).

### Combination of Gal-9 and Tim-3 expression was independent prognostic marker

Table 2 showed the multivariate analyses of factors related to patient prognosis. The univariate analysis showed that the following factors were significantly related to postoperative survival: TNM stage, venous infiltration and combination of Gal-9 and Tim-3 expression (*P*<0.001, *P* = 0.001, *P*<0.001 respectively). After adjusted by age and gender, multivariate regression analysis indicated that Gal-9 and Tim-3 co-expression and TNM stage were independent prognostic factors, but Lymph-vascular invasion was not an independent prognostic factor.

**Table 2:** Multivariate analysis for prediction of overall survival in gastric cancer.

	Relative risk(95% CI)	*P* value*
Gal-9 and Tim-3 coexpression(HSCORE)		
Gal-9≤200 & Tim-3 (+) (n = 121)	Reference	
Gal-9≤200 & Tim-3 (−) (n = 87)	0.71(0.45–1.10)	0.129
Gal-9>200 & Tim-3 (+) (n = 62)	0.60(0.35–1.01)	0.056
Gal-9>200 & Tim-3 (−) (n = 35)	0.43(0.20–0.93)	0.033
TNM stage		
Ι (n = 25)	Reference	
ΙΙ (n = 51)	1.60(0.44–5.88)	0.478
ΙΙΙ (n = 195)	3.67(1.13–11.87)	0.030
IV(n = 34)	11.98(3.53–40.74)	0.001

* Cox proportional hazards model, adjusted for gender, age,lymph-vascular invasion, TNM stage.

## Discussion

In this study, higher expressions of Gal-9 were found in patients without lymph-vascular invasion, lymph node metastasis and distant metastasis. Moreover, higher expressions of Gal-9 is closely associated with better survival. These findings are consistent with a previous report in breast cancer, hepatocellular carcinoma, cervical carcinoma and malignant melanoma [12], [13], [14], [15], [22]. Gal-9 expression in gastric cancer lesions was significantly higher than that in paired adjacent normal gastric epithelial tissues, this result led us to hypothesize that Gal-9 exhibit anti-tumor activity in tumor-bearing tissues through incresed aggregation of tumor cell [23]. However, this finding is different from the Liang's observations. They found that Gal-9 expression in cancer tissues of 38 invasive cervical squamous cell carcinoma (SCC) patients was significantly lower than that in normal cervical squamous epithelium from 23 healthy controls. But Gal-9 expressions in well differentiated SCC were significant higher compared to those in high grade squamous intrapiehelial lesions [14]. Unfortunately, they did not compare the Gal-9 expression in cancer tissues to paired adjacent normal tissues. The discrepancy from different studies may be due to individual variation of immune state,causes of inflammation and tissue specificity of Gal-9 expression. In addition, various diseases are sensitive to apoptotic elimination by recombinant Gal-9. In line with this observation, suggesting that gal-9 may be an attractive candidate for the treatment of cancer. Treatment with recombinant Gal-9 may prevent metastatic spread of cancer [16], [24], [25].

Tim-3 is the surface molecule that can specifically identify Th1 cells from Th2 cells in both mice and humans [26]. It has also been found on cytotoxic CD8+ T cells, Th17, Treg, monocytes, dendritic cells, mast cells, natural killer (NK) cells, melanoma, SCC and lung cancer cells [19], [20], [27], [28], [29], [30]. Here, for the first time, we demonstrated Tim-3 expression in gastric cancer cells and cell lines. In addition, we found a significant correlation between Tim-3 expression in cancer cells and the clinical-pathologic parameters of lymph-vascular invasion. Furthermore, we found that the survival rate of patients with positive Tim-3 expression tumors was significantly lower than those with negative Tim-3 expression. The univariate and multivariate analyses also revealed that Tim-3 overexpression in tumor cells associated with poor prognosis of gastric cancer.

Tim-3 is a powerful immunoinhibitory molecule involved in immune tolerance, and autoimmune responses. The mechanisms for regulating Tim-3 expression in tumor cells are not yet fully understood. Wiener et al reported that TGF-β1 elevated Tim-3 expression, which resulted in local immunosuppression [19]. Meanwhile, high tumor TGF-β1 levels were significantly associated with decreased survival of the gastric cancer patients. Previous studies have identified Tim-3 as a negative regulator of T helper 1 -cell responses [26]. Recently, however, new researches show that Tim-3 also can promote a pro-inflammatory response, indicating that Tim-3 has a dual role in immunity [31], [32]. Thus, the expression levels of Tim-3 in cancer cells may associate with cytokine producing during the process of gastric carcinogenesis. Moreover, recent studies have identified that Tim-3 overexpression was associated with shorter overall survival of epithelial cancers including lung cancer and cervical cancer [20], [30], our findings are consistent with these reports. More importantly than all of these findings, Cao et al confirmed that down regulating the expression of Tim-3 in Hela cells significantly inhibited both the migration and the invasion of Hela cells [30]. These results suggested that Tim-3 not only suppress anti-tumor immunity, but also directly promote cancer progress.

Identification of Gal-9 as a ligand for Tim-3 has now firmly established the Tim-3-Gal-9 pathway as an important regulator of Th1 immunity, which results in apoptosis of Th1 cells [21], [33]. In this study, we found that both higher Gal-9 expression and negative Tim-3 expression were significantly associated with better overall survival (*P* = 0.002, *P* = 0.010, respectively); the combination of Gal-9 and Tim-3 expression was independent prognostic marker in gastric cancer. These results indicate that the Tim-3-Gal-9 pathway may play an important role in gastric carcinogenesis.

In addition, *H.pylori* is a group 1 carcinogen based on World Health Organization (WHO) and International Agency for Research on Cancer (IARC) [34]. It has been generally accepted that gastric cancer is a multistep process involving a series of events such as atrophic gastritis, intestinal metaplasia, dysplasia and carcinoma. Previous reports have noted that the Tim-3-Gal-9 interaction can have a notable effect on inflammatory [26], [35], [36], [37]. We also examined whether *H.pylori* infection can effect expression levels of Gal-9 and Tim-3. However, in the current study, Gal-9 and Tim-3 expression levels were not statistically different between the *H.pylori* (+) group and the *H.pylori* (−) group in gastric cancer (*P* = 0.101, *P* = 0.565). Further investigations with additionalsamples are needed to establish the roles of Gal-9 and Tim-3 in *H.pylori*-infection.

In conclusion, this study has demonstrated the expression of Gal-9 and Tim-3 in gastric cancer. Importantly, the multivariate analyses revealed the significant role of Gal-9 and Tim-3 as an independent prognostic factor for patients with gastric cancer. Thus, the status of Gal-9 and Tim-3 could be determined on clinical examination and may have treatment value in the future [38], [39].
